# Bacteriocin Antagonistic Potentials of *Lactococcus cremoris* and *Lactococcus lactis* Isolates from Different Habitats

**DOI:** 10.1007/s12602-024-10361-w

**Published:** 2024-09-21

**Authors:** Taya Tang, Jørgen J. Leisner

**Affiliations:** Department of Veterinary and Animal Sciences, Faculty of Health and Medical Sciences, University of Copenhagen, Stigbøjlen 4, 1870 Frederiksberg C, Denmark

**Keywords:** Lactococci, Nisin, Lactococcin B, Comparative genomics, Antagonism, Niche adaptation

## Abstract

It has not been extensively examined if the ecological role of lactic acid bacterial bacteriocins may affect their distribution in different habitats and thereby impact screenings for novel variants. Further, the functionality of such variants requires additional investigation. This study investigated the distribution of bacteriocin biosynthetic gene clusters (BGCs) and bacteriocinogenic activity of *Lactococcus cremoris* and *Lactococcus lactis* from a range of different environments. Whole genome sequencing and phylogenetic analysis of fifty *L. cremoris* and *L. lactis* strains showed distinct species clustering without significant genome size differences between species or sources. Genomic screening with AntiSMASH and BAGEL4 identified several BGCs, with variation based on species but not on habitat. Deferred inhibition assays revealed pronounced activity only in strains possessing nisin or lactococcin B BGCs and not in strains with other BGCs. Several hitherto undescribed types of lactococcin BGCs appeared to be incomplete regarding genes encoding secretion and immunity. In conclusion, this study indicates that habitats did not appear to affect distribution of BGCs. Further, the antagonistic functionality of several BGCs was unclear indicating that improving applications of lactococcal bacteriocins may depend as much or more on research on increasing efficacy of well-known bacteriocins than searching for novel variants.

**Supplementary Information:**

The online version contains supplementary material available at 10.1007/s12602-024-10361-w.

## Introduction

Antagonistic interactions of lactic acid bacteria (LAB) have been extensively studied in food products whereas their wider ecological role is less well understood [1–5]. We have recently shown that although some foodborne isolates of the LAB species *Carnobacterium maltaromaticum* produce bacteriocins [6–9], such potential appears to be low among isolates from freshwater habitats where the species constitute a very minor element of the microbiota [10]. In addition, in most cases where isolates possessed biosynthetic gene clusters (BGCs) encoding bacteriocins, they appeared to be incomplete lacking genes encoding bacteriocin secretion, immunity, and/or regulation [10]. It is hypothesized that selection for bacteriocinogenic activity is not strong in such scenarios, possibly due to the lack of selective advantage in environments with strong competition from unrelated, non-sensitive microbiota, compared to related, sensitive target organisms.

Interestingly, some animal/human-associated LAB such as *Streptococcus mutans* and *Streptococcus pneumonia* possess high frequencies of bacteriocin-mediated activity. This suggests that some competitive environments support selection for antagonistic activity, which may then to a higher degree facilitate screenings for novel bacteriocins [4, 11, 12].

To further study the habitat effect on selection for bacteriocinogenic activity, we investigated the LAB species *Lactococcus cremoris* that to a large degree is confined to dairy products, and the closely related *Lactococcus lactis* with a broader distribution ranging from plant material, soil, and water to food products [13–18]. We screened genomes from isolates of the two species from different habitats for bacteriocin BGCs and tested selected isolates for the presence and spectrum of antagonistic activities. We discuss the relevance of the results for the prospect of finding new BGCs encoding functional bacteriocinogenic systems by screening isolates from different habitats.

## Materials and Methods

### Bacterial Strains

Three *L. lactis* isolates, 2B-1, 2B-5, and 2B-9, were from a water sample (pH 4.85) obtained at 5–10 cm depth along the shore of a freshwater fen situated in a forest north of Copenhagen in 2005 [19]. Additional strains of *L. cremoris* and *L. lactis* used in the antagonistic assays were obtained from the Belgian Coordinated Collections of Microorganisms (BCCM), including LMG 6890, LMG 6897, LMG 8520, LMG 8526, LMG 9447, and LMG 24662. IL1403 and MG1363 were obtained from the culture collection of Dpt. Food Science, University of Copenhagen. Target strains isolated from freshwater environments, included in the antagonistic assay, were obtained in the study by Tang et al. [10]. Additional target strains were from the culture collection at the Dpt. Veterinary and Animal Sciences, University of Copenhagen.

### Phylogenomic Analysis

The three freshwater isolates of *Lactococcus lactis* subsp. *lactis* 2B-1, 2B-5, and 2B-9 were whole genome sequenced on PromethION P24 (Oxford Nanopore Technologies) and followed by de novo assembly, quality control, classification, and annotation, using methodologies as described by Tang and Leisner [20]. Raw sequence reads have been deposited at the NCBI under BioProject with accession PRJNA1110574, BioSamples with accession SAMN41371689-SAMN41371691, and SRA with accession SRR28999492-SRR28999494. The sampling location description and genome characteristics of the three isolates are summarized in Table S1.

**Fig. 1:**
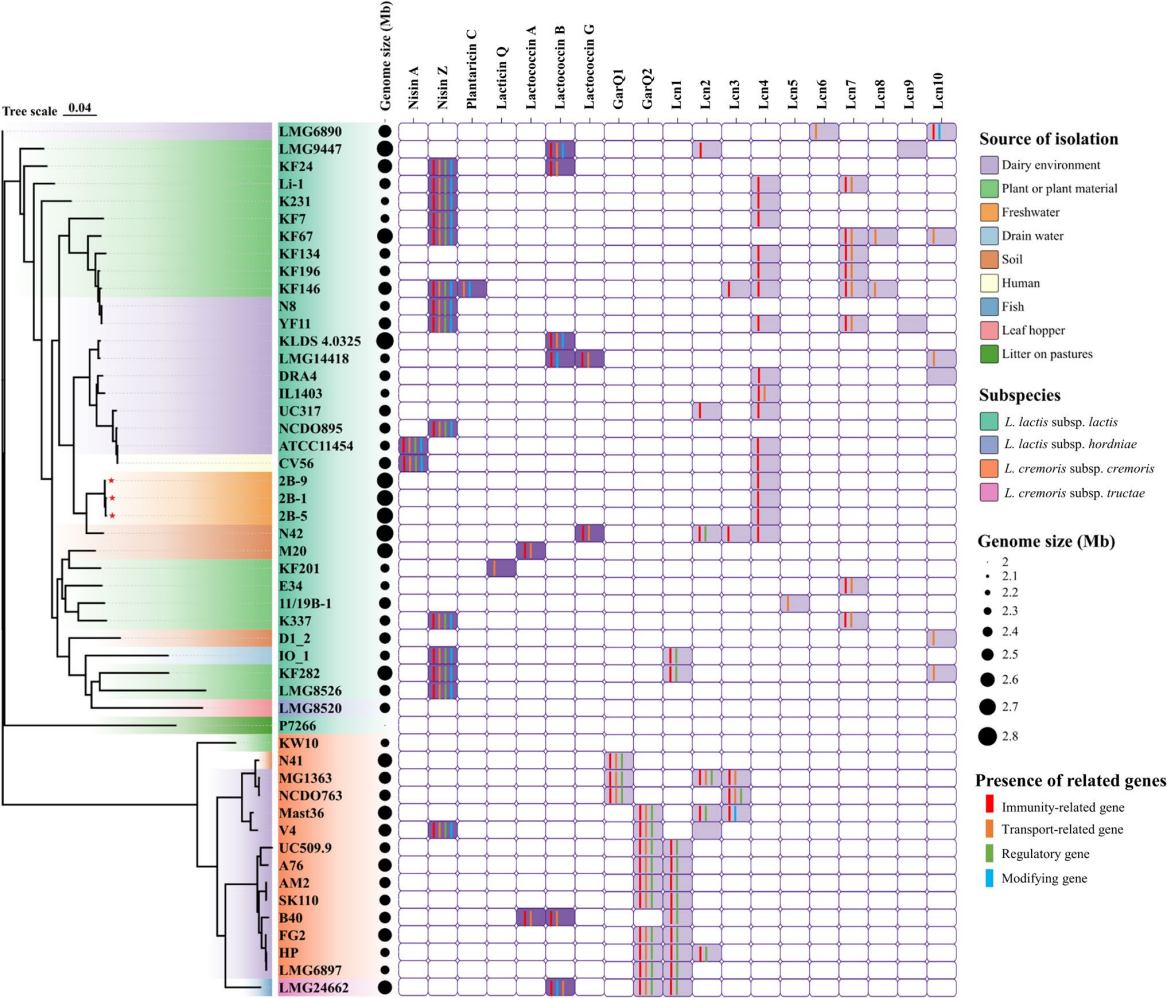
A maximum-likelihood phylogenetic tree based on SNP variants in the whole core genomes of the fifty *L. cremoris* and *L. lactis* strains was generated using CSI Phylogeny v.1.4 (Kaas et al., [21]) and visualized in tvBOT (Xie et al., [22]). The reference genome for SNP mapping and screening was *L. lactis* subsp*. lactis* LMG 6890. The branches of the tree are color-coded to represent the varied isolation sources of the strains. Three freshwater *L. lactis* isolates are indicated with a red star at the end of branches. The strain names are color-coded to denote different *L. lactis* and *L. cremoris* subspecies. The bubble plot following the strain names depicts genome sizes, with the size of black circles proportional to each genome size. In the grid plot area, dark purple squares indicate the presence of structural genes encoding known bacteriocins from *L. cremoris* and *L. lactis* strains, while the light purple squares indicate the presence of structural genes encoding garvicin Q family bacteriocins and lactococcin family bacteriocins with variants predicted in the fifty genomes. Different colored vertical lines on the grid indicate the presence of accessory genes

A phylogenetic tree based on alignment of the single-nucleotide polymorphism (SNP) variants in the whole genome sequences of the three freshwater *L. lactis* subsp. *lactis* isolates, along with forty-seven published *L. cremoris* and *L. lactis* strains retrieved from the Genbank (Table S2), was constructed via CSI Phylogeny v1.4 and visualized in tvBOT v.2.6 (Fig. 1) [21, 22]. SNPs were identified by mapping reads against the reference genome of *L. lactis* subsp. *lactis* LMG6890 (GenBank accession number: GCA_001456385.1).

Some strains showed close phylogenetic relatedness with the three freshwater isolates 2B-1, 2B-5, and 2B-9. Their genomic similarity was assessed by calculating Digital DNA-DNA hybridization (dDDH, formula d4) values using the Type Strain Genome Server (TYGS) (https://tygs.dsmz.de) with recommended settings [23]. Further confirmation of the identity between these genome sequences was provided by calculating the average nucleotide identity (ANI) based on OrthoANIu tool from EzBioCloud [24]. The ANIu and dDDH results were visualized in a heatmap using Chiplot (https://www.chiplot.online/) (Fig. S1).

### Identification of Bacteriocin BGCs

**Fig. 2:**
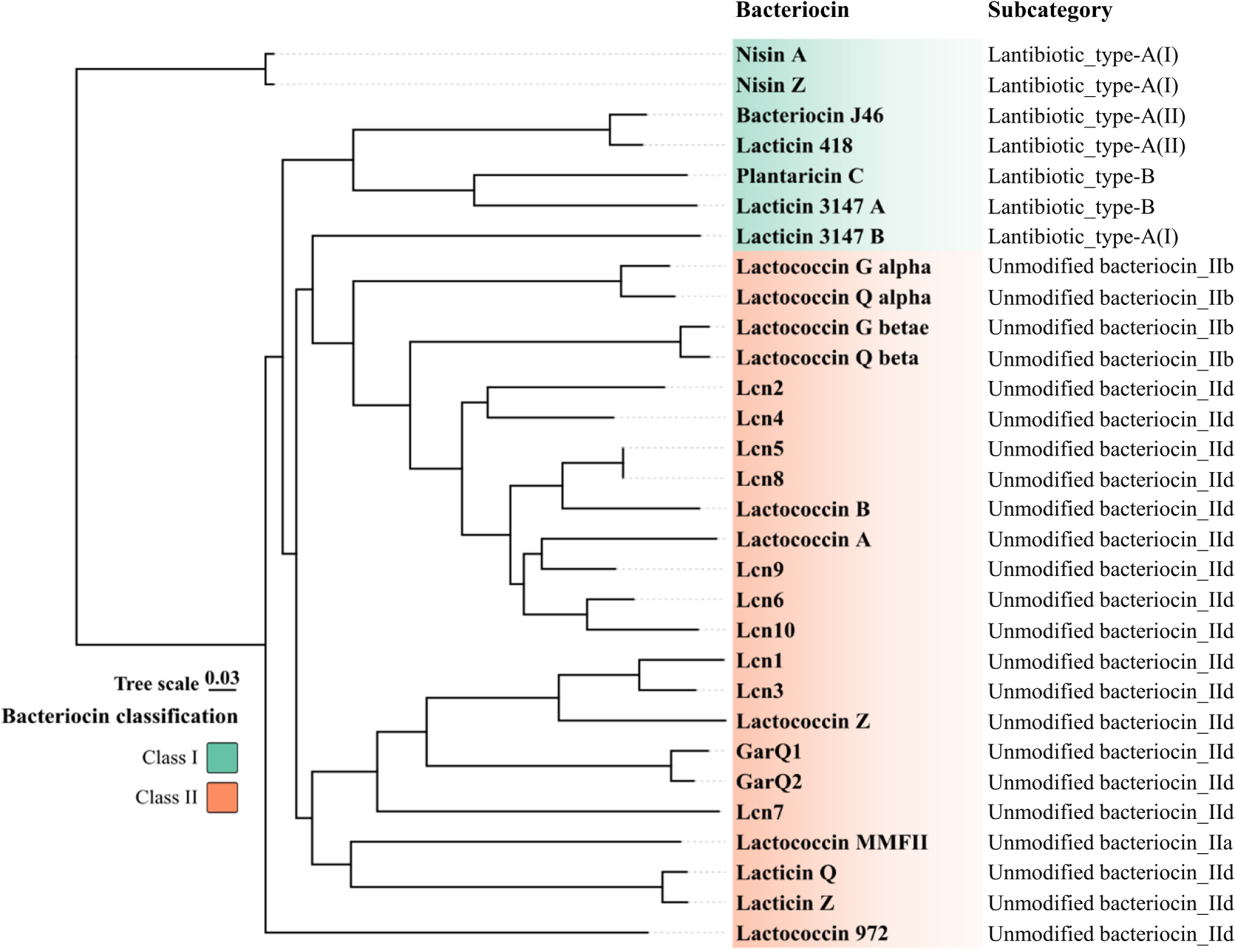
An unrooted neighbor-joining phylogenetic tree constructed based on amino acid sequences of conserved domains of various bacteriocins from *L. cremoris* and *L. lactis* genomes, including bacteriocins identified in genomes of strains analyzed in this study and additional known bacteriocin sequences from GenBank as references. See Table S2 for Genbank accession numbers for these bacteriocins, and InterPro or NCBIfam accession number for their conserved domains. See Fig. S2 for amino acid sequences alignment of domains of various bacteriocins

The presence among the fifty genomes of *L. cremoris* and *L. lactis* strains of BGCs encoding bacteriocins was predicted using AntiSMASH 7.1.0 [25] and BAGEL4 [26] with default parameters. Conserved protein domains were confirmed by the InterPro (https://www.ebi.ac.uk/interpro/) with predictive models provided by an integrated database [27], and signal peptides were further identified by SignalP 6.0 (https://services.healthtech.dtu.dk/services/SignalP-6.0/). Structural gene sequences were truncated to the length of known or predicted bacteriocin domains. Further, the amino acid sequences of the detected bacteriocin domains from the analyzed genomes were aligned with the conserved domains of other known bacteriocins from GenBank (Table S3). The sequence alignment was performed using ClustalW (Fig. S2) [28], and phylogenetic analysis of these bacteriocins based on their core domains was conducted using the neighbor-joining (NJ) method in MEGA 11 [29]. An unrooted phylogenetic tree was generated using tvBOT v.2.6 (Fig. 2) [22]. Sequence similarities between the bacteriocin domains were determined using the Basic Local Alignment Search Tool (BLAST).

### Antagonistic Assays

**Fig. 3:**
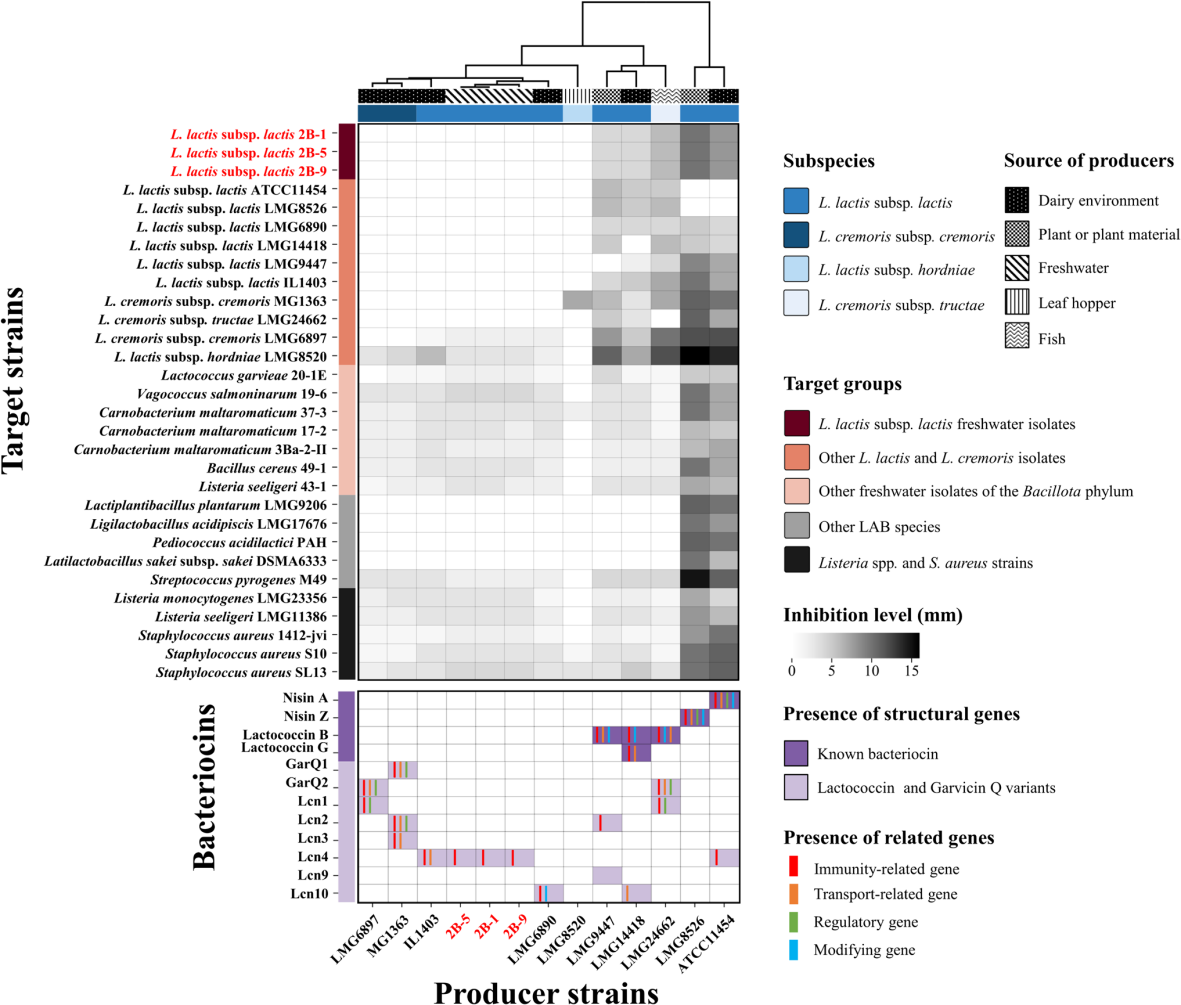
A hierarchical clustering heatmap of the antagonistic activity of thirteen *L. cremoris* and *L. lactis* strains from different sources and the presence of bacteriocin biosynthetic gene clusters (BGCs) in their genomes. The producer strains included three freshwater isolates (in red) and ten isolates from dairy, plants, fish, and leaf hopper. The heatmap illustrates the inhibitory effects of these strains against themselves, other species of the *Bacillota* phylum isolated from freshwater environment, as well as LAB reference cultures, *Listeria* spp. and *Staphylococcus aureus*. Each interaction was evaluated based on the impact of the producer strain on the growth of the target strain. White squares represent no inhibition, while shades ranging from light to dark indicate the size of the radius of the growth inhibition zones of target strains. The four clusters at the top of the heatmap displayed distinct antagonistic profiles of the producer strains. The grid plot at the bottom of the heatmap demonstrated the presence of genes encoding various bacteriocins

The deferred inhibition assay was employed to determine the antagonistic activity of thirteen selected *L. cremoris* and *L. lactis* strains from various sources, including three freshwater isolates and ten strains from dairy environment, plant or plant material, fish and leaf hopper (Fig. 3). Firstly, a pairwise antagonistic analysis for the thirteen *L. lactis* strains was conducted as previously described [10]. Briefly, producer cultures underwent at least two subcultures in All Purpose Tween (APT) broth (Difco, Sparks, MD, USA) at 25 °C for 24 h before being point-inoculated onto APT agar plates and anaerobically incubated at 25 °C for 24 h. Subsequently, 7.5 ml of an overlay of melted and cooled APT agar, pre-inoculated with 75 µl of each target culture, was added to the plates. The plates were then incubated anaerobically at 25 °C for 24 h before scoring the results.

The inhibitory effects of the thirteen producer strains were further evaluated against target bacteria of the *Bacillota* phylum from freshwater environments as well as reference cultures of LAB and *Listeria* spp. and *Staphylococcus aureus* (Fig. 3).

### Statistical Analyses

Genome size data from *L. cremoris* and *L. lactis* strains were analyzed to explore their distribution across various factors, including strain species, sources of isolation, and presence of bacteriocin BGCs. Since some of the publicly available *L. cremoris* and *L. lactis* genomes retrieved from GenBank are at the contig-level without assembled chromosomes, only total genome sizes, presumably including plasmids, were analyzed in this study. Descriptive statistics including range, median, mean, and standard deviation were calculated using GraphPad Prism 9.0. The normality of the data was assessed using Shapiro–Wilk and Kolmogorov–Smirnov tests within the software. Differences in mean genome sizes between *L. cremoris* and *L. lactis* strains were evaluated using an independent *t*-test. For bacteriocin BGCs identified commonly in strains (specifically BGCs encoding nisin Z, lactococcin B, GarQ2, and Lcn1-4, 7 and 10), separate analyses were conducted. Independent *t*-tests were used to compare genome size means between strains with and without each BGC. Additionally, one-way ANOVA was performed to compare genome size means of strains isolated across different sources. Tukey’s Honestly Significant Difference (HSD) test was employed following significant ANOVA results to identify specific source groups that differed significantly in genome size.

Data from the antagonistic assay was analyzed for variables, including the number of inhibited strains and radius of inhibition zones. Descriptive statistics such as range, mean, and standard deviation were calculated using GraphPad Prism 9.0 for each variable for each producer strain.

## Results

### Phylogenetic Analysis of *L. cremoris* and *L. lactis* Strains

Among the strains included in this study, 15 were *L. cremoris* (14 subsp. *cremoris* and one subsp. *tructae*) and 35 were *L. lactis* (34 subsp. *lactis* and one subsp. *hordniae)*. Genome assemblies were retrieved for all strains from the GenBank database (Table S2), except for the three freshwater strains sequenced as a part of this study (Table S1).

The fifty genomes were aligned based on SNP variants using *L. lactis* subsp. *lactis* LMG 6890 as a reference to root the phylogenetic tree. This alignment resulted in a clear division into two major clades corresponding to the species *cremoris* and *lactis* (Fig. 1). Additionally, the subspecies *hordniae* strain (LMG8520) clustered with the subspecies *lactis*, while the subspecies *tructae* strain (LMG24662) clustered with subspecies *cremoris* [30]. The *L. lactis* isolates originated from various environments, including dairy, plant or plant material, freshwater, drain water, soil, human, leaf hopper, and litter on pastures. In contrast, all *L. cremoris* strains, except for KW10 (from plant), N41 (from soil), and LMG24662 (from fish), were isolated from dairy environment, revealing, therefore, a source-specific cluster (Fig. 1).

Subsequently, the strain P7266 was positioned as an outgroup (Fig. 1, Table S2), consistent with previous findings [31]. It was therefore excluded from subsequent statistical analyses. In addition, the three freshwater strains exhibited a very close phylogenetic relationship, particularly strains 2B-1 and 2B-5, which displayed identical SNP profiles. The *L. lactis* subsp. *lactis* strain N42 isolated from soil exhibited the closest phylogenetic relatedness to the freshwater isolates. Subsequently, ANI and dDDH values were calculated to assess the similarity among *L. lactis* subsp. *lactis* genomes of the three freshwater isolates (2B-1, 2B-5, and 2B-9), soil isolate N42 and reference dairy isolates (ATCC11454 and LMG6890). The ANI values ranged from 98.66 to 99.95% and dDDH values from 88.20 to 100% among the six selected genomes (Fig. S1). The three freshwater strains shared high ANI values (99.90–99.95%) and dDDH values (99.90–100%) with each other. Moreover, strain N42 displayed a higher similarity to the freshwater strains compared to the two strains from dairy, with ANI values (99.20–99.23%) and dDDH values (93.70%). These results were consistent with the relationships observed in the SNP-based phylogenetic tree (Fig. 1, Fig. S1). The genomic similarity of the three freshwater isolates was also verified by their genome characteristics shown in Table S1. Therefore, the three freshwater isolates were considered a single sample in subsequent genomic statistics analyses.

**Fig. 4:**
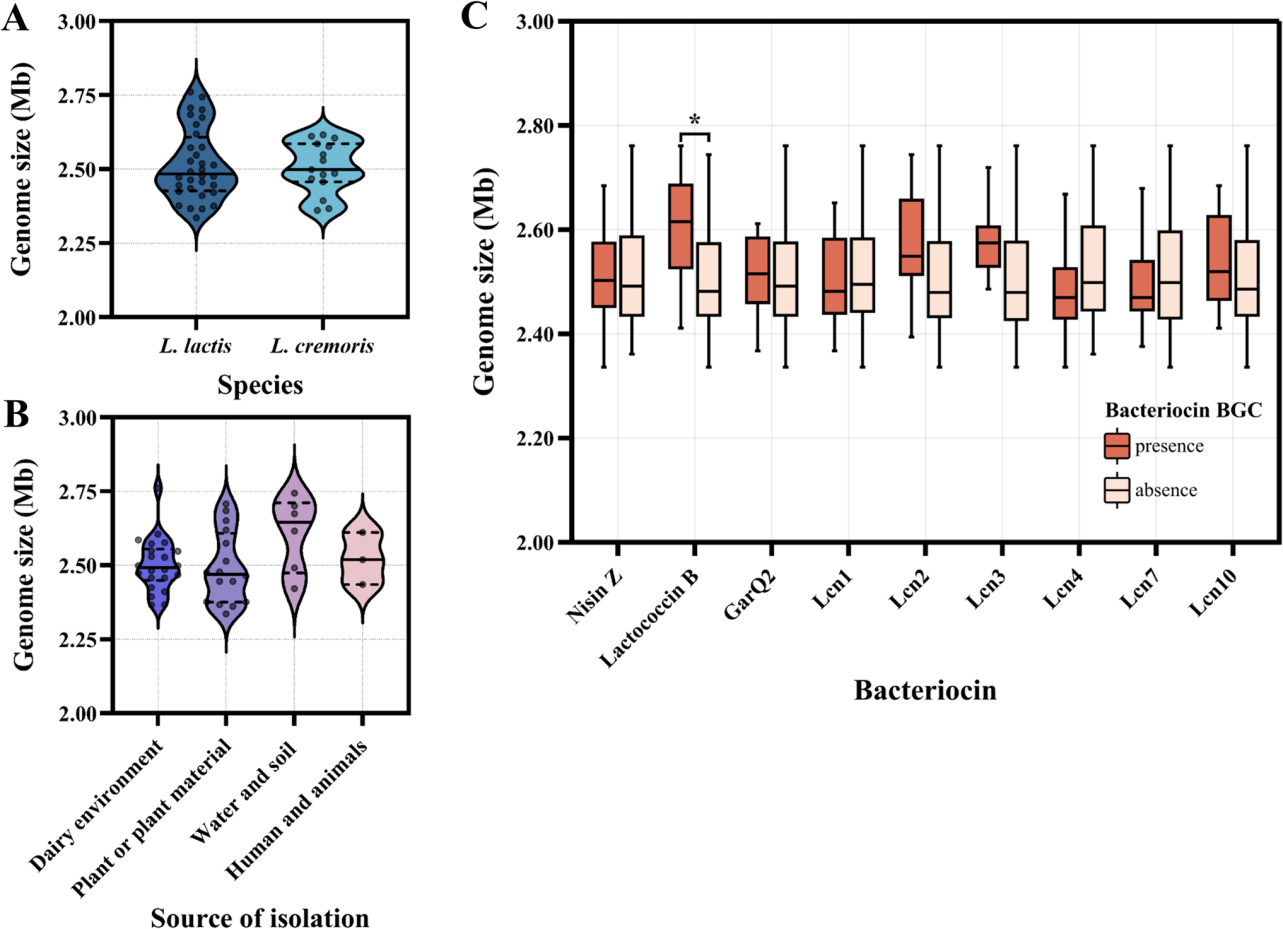
Distribution of genome sizes in 49 strains across *L. cremoris* and *L. lactis* species (**A**), different sources of isolation (**B**), and presence or absence of various bacteriocin biosynthetic gene clusters (**C**). (*p < 0.05)

The genomes of the *L. lactis* subsp. *lactis* freshwater isolates in this study were around 2.70 Mb in size, with a GC content of 35% (Fig. 1, Table S1). Across all analyzed *L. lactis* strains in this study (*n* = 32, excluding strain P7266 and lumping the three freshwater strains together), genome sizes ranged from 2.34 Mb to 2.76 Mb, with a median of 2.49 Mb and a mean of 2.52 ± 0.12 Mb. Similarly, among *L. cremoris* strains (*n* = 15), genome sizes ranged from 2.36 Mb to 2.62 Mb, with a median of 2.50 Mb and a mean of 2.51 ± 0.09 Mb. There was no significant difference in genome size means between isolates from the two *Lactococcus* species (*t*-test, *P* = 0.7511, Fig. 4, Table S4). When considering the isolation environment, all strains were grouped into four categories: dairy environment (*n* = 22) with a mean genome size of 2.50 ± 0.09 Mb, plant or plant material (*n* = 16) with a mean of 2.49 ± 0.12 Mb, water and soil (*n* = 6) with mean of 2.61 ± 0.13 Mb, and human and animals (*n* = 3) with mean of 2.52 ± 0.08 Mb. The distribution of genome size did not significantly vary across the different source environments (ANOVA, *P* = 0.1468. Figure 4, Table S4).

### Distribution of Bacteriocin Genes Among the *L. cremoris* and *L. lactis* Strains

A total of 100 biosynthetic gene clusters (BGCs) containing structural genes encoding bacteriocins were identified among all fifty genomes by combining AntiSMASH 6.0 and BAGEL 4 analyses (Fig. 1). Among the forty-seven genomes (excluding strain P7266 and lumping the three freshwater strains together), 65 out of 98 bacteriocin BGCs (66%) were detected within the *L. lactis* subsp. *lactis* strains, and 33 out of 98 bacteriocin BGCs (34%) were within the *L. cremoris* strains. Seven known bacteriocins were detected: nisin A, nisin Z, plantaricin C, lacticin Q, lactococcin A, lactococcin B, and lactococcin G (with subunits alpha and beta). Additional identified bacteriocins included two garvicin Q family bacteriocins (GarQ1 and GarQ2) and ten lactococcin family bacteriocins (Lcn1-Lcn10) listed arbitrarily (Fig. 1, Fig. 2, Table S3).

All the nisin BGCs included genes encoding accessory proteins involved in transport, immunity, modification, and regulation. Additionally, one structural gene encoding a plantaricin C family lantibiotic was identified in *L. lactis* subsp. *lactis* FK146, along with genes encoding accessory proteins involved in transport and modification, but not immunity.

For Class II unmodified bacteriocins, garvicin Q family bacteriocin BGCs were found exclusively in *L. cremoris*, containing two structural genes (*GarQ1* and *GarQ2*) along with genes encoding the corresponding immunity protein and accessory proteins involved in transport and regulation. In contrast, the BGCs encoding lacticin Q, lactococcin G (alpha & beta), and Lcn4-Lcn10 were found only in subspecies *lactis*. BGCs encoding lactococcin A and B, and Lcn1-Lcn3, were found both in *L. cremoris* and *L. lactis* strains, however, there was no consistent pattern in the presence of accessory genes among strains for these BGCs (Fig. 1). In addition, only structural genes *Lcn1*, *Lcn3*, and *Lcn7* as well as the gene encoding lactococcin 972 possessed sequences encoding sec-dependent signal peptides (Table S3).

Some bacteriocin BGCs were relatively commonly found among the strains, including nisin Z (14 out of 47 strains: 30%), lactococcin B (6 out of 47 strains: 13%), GarQ2 (10 out of 15 *L. cremoris* strains: 67%), Lcn1 (11 out of 47 strains overall: 23% and 9 out of 15 *L. cremoris* strains: 60%), Lcn2 (7 out of 47 strains: 15%), Lcn3 (5 out of 47 strains: 11%), Lcn4 (14 out of 32 *L. lactis* strains: 44%), Lcn7 (8 out of 32 *L. lactis* strains: 25%), and Lcn10 (6 out of 32 *L. lactis* strains: 19%) (Fig. 1). Independent *t*-tests indicated that only the presence of lactoccocin B BGC was significantly associated with genome sizes. Thus, strains containing this BGC (*n* = 6) had a mean genome size of 2.60 ± 0.13 Mb, compared to 2.50 ± 0.10 Mb for strains (*n* = 41) without it (*P* < 0.05, Fig. 4, Table S4). In overall, 24 out of 32 *L. lactis* strains (75%) possessed BGCs with additional genes encoding immunity protein and transport protein, including class I BGCs for 15 strains (47%) and class II BGCs for 14 strains (44%). Contrarily, for *L. cremoris*, this was the case for only one out of 15 strains (6%) for class I BGC whereas 14 out of 15 strains (93%) possessed such class II BGCs. Thus, in overall, the two species showed clear differences in distributions of BGCs, based on the bacteriocin class.

Opposed to the *L. cremoris* strains, the *L. lactis* subsp. *lactis* strains originated to a higher degree from various sources: dairy environment (*n* = 10), plant or plant material (*n* = 15), water and soil (*n* = 5, lumping the three freshwater isolates together), human and animal (*n* = 2) (Fig. 1, Table S2). The numbers of predicted bacteriocin BGCs were correlated to the number of isolates analyzed from each type of environment. Thus, isolates from plant or plant material encoded a total of 34 BGCs, followed by isolates from the dairy environment (18 BGCs), water and soil (9 BGCs), and human and animal (2 BGCs). This trend was also observed regarding the variety of bacteriocins in *L. lactis* subsp. *lactis* strains, as isolates from plant or plant material contained 13 out of 17 types of bacteriocins (76%), those from the dairy environment had 10 out of 17 types (59%), isolates from water and soil contained 8 out of 17 types (47%), and those from humans and animal had 2 out of 17 types (12%).

A phylogenetic analysis, based on the core domains of structural genes encoding bacteriocins from the genomes analyzed in this study as well as the core domains of other known bacteriocins produced by *L. cremoris* and *L. lactis* strains retrieved from public dataset, was done with the aim of examining the relationship of the distinct lactococcins Lcn1 to Lcn10 (Fig. 2, Table S3). Class I bacteriocins were clustered based on their specific domains, which could be further divided into subgroups (Fig. 2) [32]. Type-A(I) lantibiotics included nisin A and nisin Z, while type-A(II) lantibiotics included bacteriocin J46 and lacticin 418. Type-B lantibiotics were represented by plantaricin C. Lacticin 3147 formed a distinct group as a two-component lantibiotic, consisting of an alpha-peptide (lacticin 3147 A, a type-B like lantibiotic) and a beta-peptide (lacticin 3147 B, a type-A(I) like lantibiotic) [33]. Class II unmodified bacteriocins included class IIa bacteriocin possessing a conservative N-terminal YGNG motif (lactococcin MMFII), class IIb two-component bacteriocins (lactococcin G and lactococcin Q), and class IId single-peptide bacteriocins (lactococcin A, lactococcin B, lactococcin Z, lactococcin family bacteriocins Lcn1-Lcn10, garvicin Q family bacteriocins GarQ1 and GarQ2, lacticin Q and lacticin Z, and lactococcin 972) [34]. Among the lactococcin family bacteriocins, Lcn2 and Lcn4 showed 65.22% similarity in their domain sequences. Lcn5 and Lcn8 were very closely related, having identical conserved domain sequences, and sharing 77.27% similarity with the structural gene of lactococcin B. Lcn9 was closely related to lactococcin A, with 71.43% identity in their domain sequences of structural genes. Lcn6 and Lcn10 clustered together with 81.82% similarity in their domain sequences. Finally, Lcn1 and Lcn3 were closely related, with 83.78% similarity in their domain sequences, and also related to lactococcin Z, with similarities of 66.67% and 63.89%, respectively. The core domains of GarQ1 and GarQ2 showed 93.02% identity. Only Lcn7 was distinct from other class IId bacteriocins (Fig. 2).

### Antagonistic Assays

The thirteen strains of *L. cremoris* and *L. lactis,* including the three freshwater isolates from this study and ten strains from a variety of sources, exhibited four different antagonism profiles (Fig. 3, Fig. S3). ATCC11454 and LMG8526 showed the most significant growth inhibition effect against all target strains. These two strains were the only producers that contain BGC encoding nisin A or nisin Z. LMG 9447, LMG 14418, and LMG 24662 all showed significant activity towards *L. cremoris* and *L. lactis* target strains but no other target strains. The genomes of all three strains contained the lactococcin B BGC and in addition the LMG 14418 also had the lactococcin G BGC. The LMG8520 strain had the narrowest spectrum of inhibition — and the highest susceptibility — which agreed with no genes encoding antimicrobial compounds being detected in this strain. The activity of this strain toward MG1363 is not readily explained. The remaining seven strains showed limited antagonistic activity to one or two *L. cremoris* and *L. lactis* target strains and weak activity to a range of freshwater isolates belonging to *Bacillota* phylum as well as target strains of *Streptococcus pyrogenes*, *Listeria* spp., and *Staphylococcus aureus*. The inhibition zones observed were small (< 3 mm in radius) and some zones had diffuse boundaries. This weak activity may be due to acid production rather than bacteriocinogenic activity.

Among the producers, the freshwater strains (2B-1, 2B-5, and 2B-9) displayed similar inhibitory and sensitive activities in this antagonistic assay, thus they were considered as a single sample in subsequent calculation analysis. In overall, there were 197 antagonistic interactions out of 308 paired combinations (64%) exhibited in the competition network. Six out of 11 (55%) strains showed no significant antagonistic activity whereas three strains (27%) showed narrow antagonistic activity, with an average of 23 ± 0 inhibited target strains and an average inhibition zone radius ranging from 3.39 ± 1.47 mm to 4.17 ± 2.15 mm. Two strains (18%) demonstrated significant antagonistic activity, with an average of 26 ± 0 inhibited target strains and an average inhibition zone radius of 9.35 ± 2.77 mm and 7.85 ± 2.68 mm, respectively (Fig. 3, Fig. S3). The five strains with clear antagonistic activity came from sources including dairy, plant, and fish, whereas the remaining strains originated mostly from the dairy environment but also from freshwater and leaf hopper (Fig. 3).

## Discussion

The average genome sizes of isolates between the two species *L. cremoris* and *L. lactis* did not show a significant difference in this study (Fig. 1, Table S4). Similar results were observed by Laroute et al. [35], who found mean genome size was 2504 kb for *L. lactis* strains and 2547 kb for *L. cremoris* strains in an analysis of eighty-three genomes. A study by Kelly et al. [16] involving eighty strains of the two species, showed that the origin of these strains affected the chromosome length, with dairy strains of *L. cremoris* having the smallest chromosomes (mean length of 2412 kb). However, our findings, based on whole-genome analysis, revealed that the distribution of genome size did not indicate a significant adaptation to the isolation environments (Table S4). Specifically, smaller genomes were observed in both *L. cremoris* and *L. lactis* strains from non-dairy environments, with genome sizes below 2,376 kb, including *L. lactis* subsp. *lactis* strains KF7, E34, KF201, K231, and *L. cremoris* subsp. *cremoris* strain KW10 (Fig. 1, Table S2). Further, the presence of bacteriocin BGGs was not correlated with genome sizes, except to a very minor degree for lactococcin B.

The frequencies of bacteriocin-like activities among isolates of *L. cremoris* and *L. lactis* have been reported to be relatively low. Geis et al. [36] found bacteriocin activity in about 5% of 280 strains from a culture collection and commercial starter cultures. Moreno et al. [37] reported 8.4% with activity among 167 dairy-related strains, with 2% in *L. cemoris* and 12% in *L. lactis* strains. A study on cross-inhibition among 9 strains of *L. cremoris* and 23 strains of *L. lactis*, all originating from the same production of cheese with no added starter culture, showed inhibition in 12.7% of pairwise combinations [38]. These studies did not examine the phylogenies of the strains, making it difficult to evaluate the representativeness of the results.

In the present study, bacteriocin-related genes correlated with significant antagonistic activity and present in both *L. cremoris* and *L. lactis* (Fig. 3: nisin A, nisin Z, and lactococcin B) were found in 8 out of 22 (36%) dairy-associated strains and 14 out of 25 (56%, excluding strain P7266 and lumping three freshwater isolates together) non-dairy-associated strains (Fig. 1). These frequencies cover a wide phylogenetic range of strains and may reasonably represent the overall presence of nisin A, nisin Z, and lactococcin B.

Interestingly, only the presence of the nisin or Lactococcin B BGCs was associated with pronounced antagonistic activity (Fig. 3 and Fig. S3). Strains with nisin BGCs exhibited strong broad-spectrum activity, while those with lactococcin B BGCs showed strong narrow-spectrum activity, specifically targeting *L. cremoris* and *L. lactis*. This suggests that nisin and lactococcin B may have different ecological roles. For example, nisin-producing strains clearly inhibited target strains isolated from freshwater environments, whereas this was not the case to the same extent for strains with lactococcin B BGCs (Fig. 3).

Some strains that possessed BGCs with associated genes for immunity, secretion, or modification — such as lacticin Q, plantaricin C, and lactococcin G — were not tested for antagonistic activity in this study. One strain that possessed the lactococcin G BGC also possessed lactoccocin B preventing independent testing of its contribution to antagonistic activity. A strain with Lcn9 (containing only the structural gene) showed significant activity, likely due to the presence of the Lactococcin B BGC. Strains with GarQ1 and GarQ2 as well as Lcn1-Lcn4 and Lcn10 BGCs were tested but showed no antagonistic activity (Fig. 3).

The underlying reason for this remains unclear, but it may be that the standard conditions of the inhibition assay did not promote their expression [39], at least for GarQ1 and GarQ2 for which the BGCs did not appear to be incomplete (Fig. 1). Additionally, the mannose phosphotransferase system (Man-PTS), known as the receptor for class IIa bacteriocins as well as class IId bacteriocins (such as lactococcin A, lactococcin B, and garvicin Q), plays a crucial role in the susceptibility of target strains. Genetic variations in the membrane-bound proteins IIC and IID of the Man-PTS can prevent the antibacterial activity of these bacteriocins [40, 41]. Moreover, modifications to the cell membrane can hinder bacteriocin-cell interactions by altering the composition of membrane phospholipids, changing membrane fluidity, or synthesizing proteins that modify or protect the membrane [42, 43].

Most bacteriocins are initially produced as inactive precursors with an N-terminal leader peptide attached to the C-terminal core peptide. The leader peptide typically functions as a secretion signal for a dedicated transport protein or for the general secretion pathway [44]. Some class II bacteriocins, such as lactococcin 972, divergicin A, and enterocin P, possess a *sec*-type signal peptide that is cleaved during their export via the sec-dependent pathway, thereby activating the bacteriocin [45–47]. However, there are exceptions, such as leaderless bacteriocins like lacticin Q and lacticin Z, which do not undergo any post-translational modifications and become active immediately after translation [48]. Interestingly, the lactococcin family bacteriocin BGCs identified in this study, with the exception of Lcn1, Lcn3, and Lcn7, did not possess secretory signal peptides. This suggested that these bacteriocins may not be secreted by the *sec* pathway nor by dedicated secretion by associated genes [49].

Furthermore, for Lcn 5 and Lcn 8–10 BGCs, only the structural genes and transport genes were found in most cases, without genes encoding immunity or regulatory proteins. The lack of regulatory and secretory features for these BGCs obscures their biological functions, similar to what we have suggested for carnobacteriocin BM1 [10]. The BM1 BGC consists only of genes encoding the bacteriocin and the immunity protein. It is widely distributed among environmental isolates of *C. maltaromaticum* but only expressed if additional genes encoding proteins involved in regulation and secretion, associated with, e.g., the carnobacteriocin B2 BGC is present [50, 51]. One possibility, as suggested for streptococcal bacteriocinogenic systems, is that such BGCs if they include immunity proteins may enable the cell to sense incoming external bacteriocins produced by competitors and to produce immunity proteins [52]. Further studies, involving expression analyses as well as elucidation of antimicrobial activity of synthetic versions of these bacteriocins will be required to illuminate their functionality.

## Conclusion

Overall, although this study supported a species association for some of the bacteriocins examined, there appeared to be no association between niche and presence of specific bacteriocins. This lack of niche association has also been noted for type strains for species of *Lactobacillus* sensu lato [53] and suggests that different environments, at least in these cases, do not exert varying selection pressures for antagonistic activity. Further experimental studies on lactococci bacteriocins similar to those reported for *Limosilactobacillus reuteri* producing the tetramic acid derivative reutericyclin [4] are required to understand their relevance for competitiveness in various habitats. However, given the distinct properties and modes of action of bacteriocins compared to reutericyclin, specific experimental conditions should be tailored to the properties and mechanisms of the bacteriocins.

Our screening showed that only isolates that possessed BGCs encoding nisin and lactococcin B, two well-known types of bacteriocins, exhibited strong antagonistic activity. The presence of several additional BGCs encoding presumptive bacteriocins was not correlated with such activity, which in most cases could be explained as a result of incomplete BGCs. It may be possible to examine the potential antagonistic activity of bacteriocins encoded in such BGCs by cell-free synthesis using gene libraries [54]. Moreover, bacteriocin intra-species susceptibility varies significantly [55, 56], which presents a challenge in accurately defining their antagonistic activities. Thus, including a broader and more diverse range of representative target strains in future studies could provide a more comprehensive evaluation of bacteriocin activity.

In overall, the current limited understanding of the antagonistic functionality of such BGC variants, including Lcn1-Lcn10, indicates that improved applications of lactococci bacteriocins may depend more on focusing on well-known bacteriocins like nisin. This can be achieved by optimizing production conditions, refining purification techniques, combining bacteriocins with other antimicrobial agents and utilizing genetic engineering and nanotechnology [57–60]. This approach may at least in the short run be more effective than searching for novel variants, as has also been suggested for LAB bacteriocins in general [3].

## Supplementary Information

Below is the link to the electronic supplementary material.

Supplementary file1 (DOCX 2.34 MB)

## Data Availability

Sequence data that support the findings of this study have been deposited at the NCBI under BioProject with accession PRJNA1110574, BioSamples with accession SAMN41371689-SAMN41371691, and SRA with accession SRR28999492-SRR28999494.
