# Supplementary material for: Bacteriocin Antagonistic Potentials of Lactococcus cremoris and Lactococcus lactis Isolates from Different Habitats
Source: Probiotics Antimicrob Proteins. 2024 Sep 21;17(6):4203–14. doi: 10.1007/s12602-024-10361-w (PMC12634713; doi:10.1007/s12602-024-10361-w)
Supplement: Supplementary file 1 — Supplementary file1 (DOCX 2.34 MB) [file 12602_2024_10361_MOESM1_ESM.docx]

**Table S1.** Sample location and genome characteristics for *L. lactis* subsp. *lactis* freshwater isolates

|  | **2B-1** | **2B-5** | **2B-9** |
| --- | --- | --- | --- |
| **Description of location** | | | |
| Location | Copenhagen, Northern suburb, Store Hareskov | | |
| Type | Freshwater fen | | |
| pH | 4.85 | | |
| Geographic coordinates | 55°46'12.2"N 2°24'29.8"E | | |
| **Sequencing statistics** | | | |
| Number of raw reads (Mbp) | 15109 | 9997 | 5262 |
| Raw-read N50 (bp) | 10717 | 10526 | 10696 |
| Quality score of raw reads | 20.4 | 20.4 | 20.4 |
| Number of filtered reads (Mbp) | 2347 | 1521 | 1448 |
| Filtered-read N50 (bp) | 9983 | 9745 | 10425 |
| Quality score of filtered reads | 24.0 | 24.0 | 23.2 |
| **Genome statistics** | | | |
| Assembly size (bp) | 2700706 | 2700704 | 2700707 |
| Number of circular contigs | 2 | 2 | 2 |

| Chromosome size (bp) | 2623330 | 2623329 | 2623332 |
| --- | --- | --- | --- |
| Plasmid size (bp) | 77376 | 77375 | 77375 |

| Contig N50 (bp) | 2623330 | 2623329 | 2623332 |
| --- | --- | --- | --- |
| Contig L50 | 1 | 1 | 1 |
| GC (%) | 35.0 | 35.0 | 35.0 |
| Genome coverage | 868× | 562× | 529× |
| Total number of CDS | 2683 | 2675 | 2654 |
| Number of 5S rRNA | 7 | 7 | 7 |
| Number of 16S rRNA | 6 | 6 | 6 |
| Number of 23S rRNA | 6 | 6 | 6 |
| Number of tRNAs | 72 | 72 | 72 |
| Completeness (%)^a^ | 99.98 | 100.00 | 100.00 |
| Contamination (%)^b^ | 1.98 | 2.78 | 1.73 |
| GTDB Taxonomy^c^ | *L. lactis* subsp. *lactis* | *L. lactis* subsp. *lactis* | *L. lactis* subsp. *lactis* |
| **Data accession** | | | |
| BioProject | [PRJNA1110574](https://www.ncbi.nlm.nih.gov/bioproject/PRJNA1110574) | [PRJNA1110574](https://www.ncbi.nlm.nih.gov/bioproject/PRJNA1110574) | [PRJNA1110574](https://www.ncbi.nlm.nih.gov/bioproject/PRJNA1110574) |
| BioSample | [SAMN41371689](https://www.ncbi.nlm.nih.gov/biosample/?term=SAMN41371689) | [SAMN413716890](https://www.ncbi.nlm.nih.gov/biosample/?term=SAMN41371690) | [SAMN41371691](https://www.ncbi.nlm.nih.gov/biosample/?term=SAMN41371691) |
| Chromosome^d^ | [CP157495](https://www.ncbi.nlm.nih.gov/nuccore/CP157495.1) | [CP157498](https://www.ncbi.nlm.nih.gov/nuccore/CP157498.1) | [CP157500](https://www.ncbi.nlm.nih.gov/nuccore/CP157500.1) |

| Plasmid^d^ | [CP157496](https://www.ncbi.nlm.nih.gov/nuccore/CP157496.1) | [CP157497](https://www.ncbi.nlm.nih.gov/nuccore/CP157497.1) | [CP157499](https://www.ncbi.nlm.nih.gov/nuccore/CP157499.1) |
| --- | --- | --- | --- |

| SRA^e^ | [SRR28999492](https://www.ncbi.nlm.nih.gov/sra/?term=SRR28999492) | [SRR28999493](https://www.ncbi.nlm.nih.gov/sra/?term=SRR28999493) | [SRR28999494](https://www.ncbi.nlm.nih.gov/sra/?term=SRR28999494) |
| --- | --- | --- | --- |

^a^ Completeness (%) is the estimated genome completeness based on the presence and/or absence of essential lineage-specific marker genes.

^b^ Contamination (%) is the estimated genome contamination based on the presence of multiple single-copy marker genes.

^c^ GTDB Taxonomy denotes the highest taxonomy assigned to specific genomes when classified against the Genome Taxonomy Database (GTDB).

^d^ The genome of each strain was assembled into two circular contigs, consisting of a chromosome and a plasmid, each assigned an individual GenBank accession number.

^e^ The raw sequencing data (before quality filtering) has been deposited in the Sequence Read Archive at NCBI.

**Table S2.** Overview of the 47 *L. cremoris* and *L. lactis* strains used in phylogenetic analysis.

| Strain | Subspecies  (genotype) | Subspecies  (phenotype) | Genbank Accession no. | Genome size (bp) | Source of isolation | Type of source | Country of isolation |
| --- | --- | --- | --- | --- | --- | --- | --- |
| A76 | *cremoris* | *cremoris* | GCA_000236475.1 | 2,577,104 | Cheese factory | Dairy environment | - |
| UC509_9 | *cremoris* | *cremoris* | GCA_000312685.1 | 2,457,347 | Dairy starter | Dairy environment | Ireland |
| AM2 | *cremoris* | *cremoris* | GCA_001622405.1 | 2,481,572 | Dairy starter | Dairy environment | Netherlands |
| FG2 | *cremoris* | *cremoris* | GCA_001622365.1 | 2,586,136 | Dairy starter | Dairy environment | Netherlands |
| HP | *cremoris* | *cremoris* | GCA_001622215.1 | 2,393,955 | Dairy starter | Dairy environment | Netherlands |
| LMG6897 | *cremoris* | *cremoris* | GCA_001622295.1 | 2,367,195 | Dairy starter | Dairy environment | Netherlands |
| SK110 | *cremoris* | *cremoris* | GCA_001622375.1 | 2,467,613 | Dairy starter | Dairy environment | Netherlands |
| B40 | *cremoris* | *cremoris* | GCA_001622315.1 | 2,498,465 | Scandinavian ropy milk | Dairy environment | Netherlands |
| Mast36 | *cremoris* | *cremoris* | GCA_000969475.1 | 2,605,340 | Milk from a bovine mastitis case | Dairy environment | Finland |
| MG1363 | *cremoris* | *lactis* | GCA_000009425.1 | 2,529,478 | Dairy starter | Dairy environment | - |
| NCDO763 | *cremoris* | *lactis* | GCA_001622385.1 | 2,485,686 | Dairy starter | Dairy environment | Netherlands |
| V4 | *cremoris* | *lactis* | GCA_001622225.1 | 2,548,952 | Raw sheep milk | Dairy environment | Netherlands |
| N41 | *cremoris* | *lactis* | GCA_001622285.1 | 2,615,712 | Soil and grass | Soil | Netherlands |
| KW10 | *cremoris* | *lactis* | GCA_001622205.1 | 2,361,020 | Kaanga wai | Plant or plant material | Netherlands |
| LMG8520 | *hordniae* | *lactis* | GCA_001456625.1 | 2,435,575 | Leaf hopper | Leaf hopper | United States |
| ATCC11454 | *lactis* | *lactis* | GCA_009885055.1 | 2,424,658 | Cheese product | Dairy environment | United States |
| LMG14418 | *lactis* | *lactis* | GCA_001456695.1 | 2,410,930 | Bovine milk | Dairy environment | Belgium |
| N8 | *lactis* | *lactis* | GCA_014884605.1 | 2,573,129 | Cheese product | Dairy environment | Finland |
| YF11 | *lactis* | *lactis* | GCA_000348965.1 | 2,527,313 | Dairy | Dairy environment | China |
| LMG6890 | *lactis* | *lactis* | GCA_001456385.1 | 2,547,291 | Dairy starter | Dairy environment | Denmark |
| IL1403 | *lactis* | *lactis* | GCA_003722275.1 | 2,365,672 | Dairy starter | Dairy environment | France |
| NCDO895 | *lactis* | *lactis* | GCA_001456865.1 | 2,473,055 | Dairy starter | Dairy environment | United Kingdom |
| UC317 | *lactis* | *lactis* | GCA_001456855.1 | 2,498,421 | Dairy starter | Dairy environment | Ireland |
| KLDS 4.0325 | *lactis* | *lactis* | GCA_000479375.3 | 2,760,872 | Koumiss | Dairy environment | China |
| IO_1 | *lactis* | *lactis* | GCA_000344575.1 | 2,421,471 | Drain water | Drain water | Japan |
| D1_2 | *lactis* | *lactis* | GCA_029690025.2 | 2,491,562 | Peat swamp forests | Freshwater | Malaysia |
| N42 | *lactis* | *lactis* | GCA_001456835.1 | 2,743,919 | Soil and grass | Soil | Netherlands |
| KF67 | *lactis* | *lactis* | GCA_001456545.1 | 2,684,299 | Grapefruit juice | Plant or plant material | New Zealand |
| 11/19B-1 | *lactis* | *lactis* | GCA_013423365.1 | 2,513,929 | Surface of kiwifruit | Plant or plant material | Japan |
| CV56 | *lactis* | *lactis* | GCA_000192705.1 | 2,518,737 | Human vagina | Human | China |
| Li-1 | *lactis* | *lactis* | GCA_001456775.1 | 2,475,932 | Grass | Plant or plant material | Belgium |
| KF134 | *lactis* | *lactis* | GCA_001456725.1 | 2,463,397 | Alfalfa and radish | Plant or plant material | New Zealand |
| KF146 | *lactis* | *lactis* | GCA_001456575.1 | 2,574,519 | Alfalfa and radish | Plant or plant material | New Zealand |
| KF24 | *lactis* | *lactis* | GCA_001456505.1 | 2,619,218 | Alfalfa sprouts | Plant or plant material | New Zealand |
| KF7 | *lactis* | *lactis* | GCA_001456495.1 | 2,366,759 | Alfalfa sprouts | Plant or plant material | New Zealand |
| LMG8526 | *lactis* | *lactis* | GCA_001456655.1 | 2,477,489 | Chinese radish | Plant or plant material | United Kingdom |
| LMG9447 | *lactis* | *lactis* | GCA_001456685.1 | 2,707,537 | Frozen peas | Plant or plant material | United Kingdom |
| KF196 | *lactis* | *lactis* | GCA_001456595.1 | 2,445,893 | Japanese kaiware shoots | Plant or plant material | New Zealand |
| KF282 | *lactis* | *lactis* | GCA_001456615.1 | 2,651,253 | Mustard and cress | Plant or plant material | New Zealand |
| E34 | *lactis* | *lactis* | GCA_001456455.1 | 2,375,664 | Silage | Plant or plant material | Netherlands |
| KF201 | *lactis* | *lactis* | GCA_001456755.1 | 2,376,387 | Sliced mixed vegetables | Plant or plant material | New Zealand |
| K231 | *lactis* | *lactis* | GCA_001456475.1 | 2,336,037 | White kimchi | Plant or plant material | Japan |
| K337 | *lactis* | *lactis* | GCA_001456525.1 | 2,445,522 | White kimchi | Plant or plant material | Japan |
| P7266 | *lactis* | *lactis* | GCA_001622305.1 | 2,000,149 | Litter on pastures | Litter on pastures | Netherlands |
| DRA4 | *lactis* | *lactis* biovar *diacetylactis* | GCA_001622235.1 | 2,457,554 | Dairy starter | Dairy environment | Netherlands |
| M20 | *lactis* | *lactis* biovar *diacetylactis* | GCA_001456785.1 | 2,674,319 | Soil | Soil | Netherlands |
| LMG24662 | *tructae* | *cremoris* | GCA_002441825.1 | 2,611,420 | Brown trout | Fish | Spain |

**Table S3.** Amino acid sequences of conserved domains and signal peptides in known and predicted bacteriocins produced by *L. cremoris* ans *L. lactis* strains.

| Bacteriocin | Classifi-cation | Source organism | Genbank accession | Peptide size | Domain | InterPro or  NCBIfam  Accession ^a^ | Domain size | AA sequences of conserved domain | Signal peptide^d^ | AA sequences of signal peptide |
| --- | --- | --- | --- | --- | --- | --- | --- | --- | --- | --- |
| Nisin A^b^ | Class I | *L. lactis* | AAA88606 | 57aa | *Lantibiotic_typ-A_Bacillales* | IPR006079 | 51aa | MSTKDFNLDLVSVSKKDSGASPRITSISLCTPGCKTGALMGCNMKTATCHC | None | - |
| Nisin Z^b^ | Class I | *L. lactis* | CAA43440.1 | 57aa | *Lantibiotic_typ-A_Bacillales* | IPR006079 | 51aa | MSTKDFNLDLVSVSKKDSGASPRITSISLCTPGCKTGALMGCNMKTATCNC | None | - |
| Plantaricin C ^b^ | Class I | *L. lactis* | WP_058223819.1 | 66aa | *Lantibiotic_alpha* | IPR029243 | 26aa | SCSILSITLGNNGWICTWTAECQKGC | None | - |
| Lacticin 481^c^ | Class I | *L. lactis* | CAA50534.1 | 51aa | *Lantibiotic_typ-A_Lactobact* | IPR007682 | 50aa | MKEQNSFNLLQEVTESELDLILGAKGGSGVIHTISHECNMNSWQFVFTCC | None | - |
| Bacteriocin J46^c^ | Class I | *L. cremoris* | CAA61674.1 | 51aa | *Lantibiotic_typ-A_Lactobact* | IPR007682 | 50aa | MKEQNSFNLLQEVTESELDLILGAKGGSGVIHTISHEVIYNSWNFVFTCC | None | - |
| Lacticin 3147 A^c^ | Class I | *L. lactis* | AAF32256.1 | 59aa | *Lantibiotic_alpha* | IPR029243 | 32aa | GACSTNTFSLSDYWGNNGAWCTLTHECMAWCK | None | - |
| Lacticin 3147 B^c^ | Class I | *L. lactis* | AAF32257.1 | 65aa | *Lant_II_LchA2* | NF038161 | 51aa | QLGKYLEDDMIELAEGDESHGGTTPATPAISILSAYISTNTCPTTKCTRAC | None | - |
| Lactococcin A^b^ | Class II | *L. cremoris* | AAA25163.1 | 75aa | *Bacteriocin_IId* | IPR007464 | 60aa | MKNQLNFNIVSDEELSEANGGKLTFIQSTAAGDLYYNTNTHKYVYQQTQNAFGAAANTIV | None | - |
| Lactococcin B^b^ | Class II | *L.cremoris* | AAB22372.1 | 68aa | *Bacteriocin_IId* | IPR007464 | 60aa | MKNQLNFNIVSDEELAEVNGGSLQYVMSAGPYTWYKDTRTGKTICKQTIDTASYTFGVMA | None | - |
| Lactococcin G alpha^b^ | Class II | *L.lactis* | ACR43769.1 | 54aa | *Alpha_enterocin/lactococcin* | IPR012950 | 51aa | KELSEKELRECVGGGTWDDIGQGIGRVAYWVGKAMGNMSDVNQASRINRKK | None | - |
| Lactococcin G beta^b^ | Class II | *L.lactis* | ACR43770.1 | 60aa | *Bacteriocin_lactococcin-G* | IPR021089 | 61aa | MKNNNNFFKGMEIIEDQELVSITGGKKWGWLAWVDPAYEFIKGFGKGAIKEGNKDKWKNI | None | - |
| Lactococcin Q alpha^c^ | Class II | *L. lactis* | BAS54008.1 | 54aa | *Alpha_enterocin/lactococcin* | IPR012950 | 51aa | KELSEKELRECVGGSIWGDIGQGVGKAAYWVGKAMGNMSDVNQASRINRKK | None |  |
| Lactococcin Q beta^c^ | Class II | *L. lactis* | BAS54009.1 | 61aa | *Bacteriocin_lactococcin-G* | IPR021089 | 61aa | MKNNNNNFFKDMEIIEDQELVSITGGKKWGWLAWVEPAGEFLKGFGKGAIKEGNKDKWKNI | None | - |
| Lactococcin Z^c^ | Class II | *L. lactis* | BAU29928.1 | 70aa | *Bacteriocin_IId* | IPR007464 | 56aa | LNLFQSLDDEQLGKITGGALQFIQNTASGALYYNTKTHKYQYQQTSGAMGAAINVF | None | - |
| Lactococcin MMFII^c^ | Class II | *L. lactis* | P83002.1 | 37aa | *Bacteriocin_IIa* | IPR002633 | 18aa | YGNGVHCNKSKCWIDVSE | None | - |
| Lactococcin 972^c^ | Class II | *L. lactis* | AAF64054.1 | 91aa | *Lactococcin_972* | IPR006540 | 91aa | MKTKSLVLALSAVTLFSAGGIVAQAEGTWQHGYGVSSAYSNYHHGSKTHSATVVNNNTGRQGKDTQRAGVWAKATVGRNLTEKASFYYNFW | Sec/SPI | MKTKSLVLALSAVTLFSAGGIVAQA |
| Lacticin Q^b^ | Class II | *L. lactis* | BAF57910.1 | 53aa | *Bacteriocin_II_aureocin-like* | IPR020968 | 52aa | MAGFLKVVQLLAKYGSKAVQWAWANKGKILDWLNAGQAIDWVVSKIKQILGI | None | - |
| Lacticin Z^c^ | Class II | *L. lactis* | BAF75975.1 | 53aa | *Bacteriocin_II_aureocin-like* | IPR020968 | 52aa | MAGFLKVVQILAKYGSKAVQWAWANKGKILDWINAGQAIDWVVEKIKQILGI |  | - |
| GarQ1^b^ | Class II | *L. cremoris* | CAL97449.1 | 140aa | *GarQ_core* | NF033837 | 43aa | MNGANGYLAYDNWNKKYVYHVTKDPVSAVAGVLANGWGSAGAG | None | - |
| GarQ2^b^ | Class II | *L. cremoris* | AEU40035.1 | 74aa | *GarQ_core* | NF033837 | 43aa | MNSSNGYLAYDNWNKKYMYHVTKDPVSAVAGVLANGWGSAGAG | None | - |
| Lcn1^b^ | Class II | *L. lactis* | WP_011676572.1 | 109aa | *Bacteriocin_IId* | IPR007464 | 37aa | NLTFIQNTASGVLYYDQVNHKYVFAQTRGPMGAAIYV | Sec/SPI | MKPKQIYVILIALAVFSEFYSISVSA |
| Lcn2^b^ | Class II | *L. lactis* | WP_316419271.1 | 58aa | *Bacteriocin_IId* | IPR007464 | 28aa | MEIQTNFQIISDEELSEIVGGGYPNNQS | None | - |
| Lcn3^b^ | Class II | *L. lactis* | WP_228766080.1 | 109aa | *Bacteriocin_IId* | IPR007464 | 37aa | HLTYIQNTGSGVLYYDRVNHKYVFSQTRGAMGAAIYV | Sec/SPI | MRKNKTKFIAFALASAVIAVSYSTAASA |
| Lcn4^b^ | Class II | *L. lactis* | WP_260314555.1 | 55aa | *Bacteriocin_IId* | IPR007464 | 23aa | MENRLNFEAISDDELAKIVGGGY | None | - |
| Lcn5^b^ | Class II | *L. lactis* | GFO78185.1 | 91aa | *Bacteriocin_IId* | IPR007464 | 22aa | ENQLNFEVVSDEELAEVSGGYL | None | - |
| Lcn6^b^ | Class II | *L. lactis* | SCW67755.1 | 44aa | *Bacteriocin_IId* | IPR007464 | 22aa | MKNQLNFEVVSDEELLTTSGGQ | None | - |
| Lcn7^b^ | Class II | *L. lactis* | WP_251918216.1 | 113aa | *Lactococcin* | PF04369 | 52aa | FSNVEFQGGSTVNGWYFWMNSGTYTIFRNKYTGEYRTIQTQGTISYMLGVIA | Sec/SPI | MEVFMKKMKIKFITVALASTIIAVGYSAPASA |
| Lcn8^b^ | Class II | *L. lactis* | MCT1226956.1 | 78aa | *Bacteriocin_IId* | IPR007464 | 22aa | ENQLNFEVVSDEELAEVSGGYL | None | - |
| Lcn9^b^ | Class II | *L. lactis* | WP_017865277.1 | 49aa | *Bacteriocin_IId* | IPR007464 | 21aa | MENQTNFEVVSDEELSKINGG | None | - |
| Lcn10^b^ | Class II | *L. lactis* | WP_336255538.1 | 45aa | *Bacteriocin_IId* | IPR007464 | 35aa | MKNQLNFEVVSDEELMIINGGQNMSMTDGGFEWVY | None | - |

^a^ InterPro combines 13 protein signature databases into a single searchable resource. Therefore, detected domains were primarily referenced using InterPro accessions. If unavailable, NCBIfam accession numbers were used instead.

^b^ Gene sequences encoding these bacteriocins were detected in the fifty genomes of *Lactococcus* strains analyzed in this study.

^c^ Gene sequences encoding these bacteriocins were retrieved from the Genbank as references.

^d^ Sec/SPI: "standard" secretory signal peptides transported by the Sec translocon and cleaved by Signal Peptidase I (*Lep*).

**Table S4.** Distribution of genome sizes in *L. cremoris* and *L. lactis* strains according to species, sources of isolation and presence or absence of various bacteriocin biosynthetic gene clusters (BGCs).

| **Group** | | | **Number of strains** |  | **Genome size (Mbp)** | | | | | **T test or one-way ANOVA** | |
| --- | --- | --- | --- | --- | --- | --- | --- | --- | --- | --- | --- |
|  |  |  |  | Minimum | Maximum | Range | Median | Mean | SD | P value | Statistical difference |
| Species | *L. cremoris* | | 15 | 2.36 | 2.62 | 0.25 | 2.50 | 2.51 | 0.09 | 0.7511 | ns |
|  | *L. lactis* | | 32 | 2.34 | 2.76 | 0.42 | 2.49 | 2.52 | 0.12 |  |  |
| Source | Dairy environment | | 22 | 2.37 | 2.76 | 0.40 | 2.49 | 2.50 | 0.09 | 0.1468 | ns |
|  | Plant or plant material | | 16 | 2.34 | 2.71 | 0.37 | 2.47 | 2.49 | 0.12 |  |  |
|  | Water and soil | | 6 | 2.42 | 2.74 | 0.32 | 2.65 | 2.61 | 0.13 |  |  |
|  | Human and animals | | 3 | 2.44 | 2.61 | 0.18 | 2.52 | 2.52 | 0.09 |  |  |
| Presence of bacteriocin BGCs | Nisin Z | + | 14 | 2.34 | 2.68 | 0.35 | 2.50 | 2.51 | 0.10 | 0.9737 | ns |
|  |  | - | 33 | 2.36 | 2.76 | 0.40 | 2.49 | 2.51 | 0.11 |  |  |
|  | Lactococcin B | + | 6 | 2.41 | 2.76 | 0.35 | 2.62 | 2.60 | 0.13 | 0.0334 | * |
|  |  | - | 41 | 2.34 | 2.74 | 0.41 | 2.48 | 2.50 | 0.10 |  |  |
|  | GarQ2 | + | 10 | 2.37 | 2.61 | 0.24 | 2.52 | 2.51 | 0.09 | 0.9068 | ns |
|  |  | - | 37 | 2.34 | 2.76 | 0.42 | 2.49 | 2.51 | 0.12 |  |  |
|  | Lcn1 | + | 11 | 2.37 | 2.65 | 0.28 | 2.48 | 2.50 | 0.09 | 0.6805 | ns |
|  |  | - | 36 | 2.34 | 2.76 | 0.42 | 2.50 | 2.52 | 0.12 |  |  |
|  | Lcn2 | + | 7 | 2.39 | 2.74 | 0.35 | 2.55 | 2.58 | 0.12 | 0.1048 | ns |
|  |  | - | 40 | 2.34 | 2.76 | 0.42 | 2.48 | 2.50 | 0.11 |  |  |
|  | Lcn3 | + | 5 | 2.49 | 2.74 | 0.26 | 2.58 | 2.59 | 0.10 | 0.1085 | ns |
|  |  | - | 42 | 2.34 | 2.76 | 0.42 | 2.48 | 2.50 | 0.11 |  |  |
|  | Lcn4 | + | 14 | 2.34 | 2.74 | 0.41 | 2.47 | 2.49 | 0.12 | 0.4093 | ns |
|  |  | - | 33 | 2.36 | 2.76 | 0.40 | 2.50 | 2.52 | 0.11 |  |  |
|  | Lcn7 | + | 8 | 2.38 | 2.68 | 0.31 | 2.47 | 2.50 | 0.10 | 0.6908 | ns |
|  |  | - | 39 | 2.34 | 2.76 | 0.42 | 2.50 | 2.52 | 0.11 |  |  |
|  | Lcn10 | + | 6 | 2.41 | 2.68 | 0.27 | 2.52 | 2.54 | 0.11 | 0.5214 | ns |
|  |  | - | 41 | 2.34 | 2.76 | 0.42 | 2.49 | 2.51 | 0.11 |  |  |

ns: No significant differences. *: Significant difference (p < 0.05).


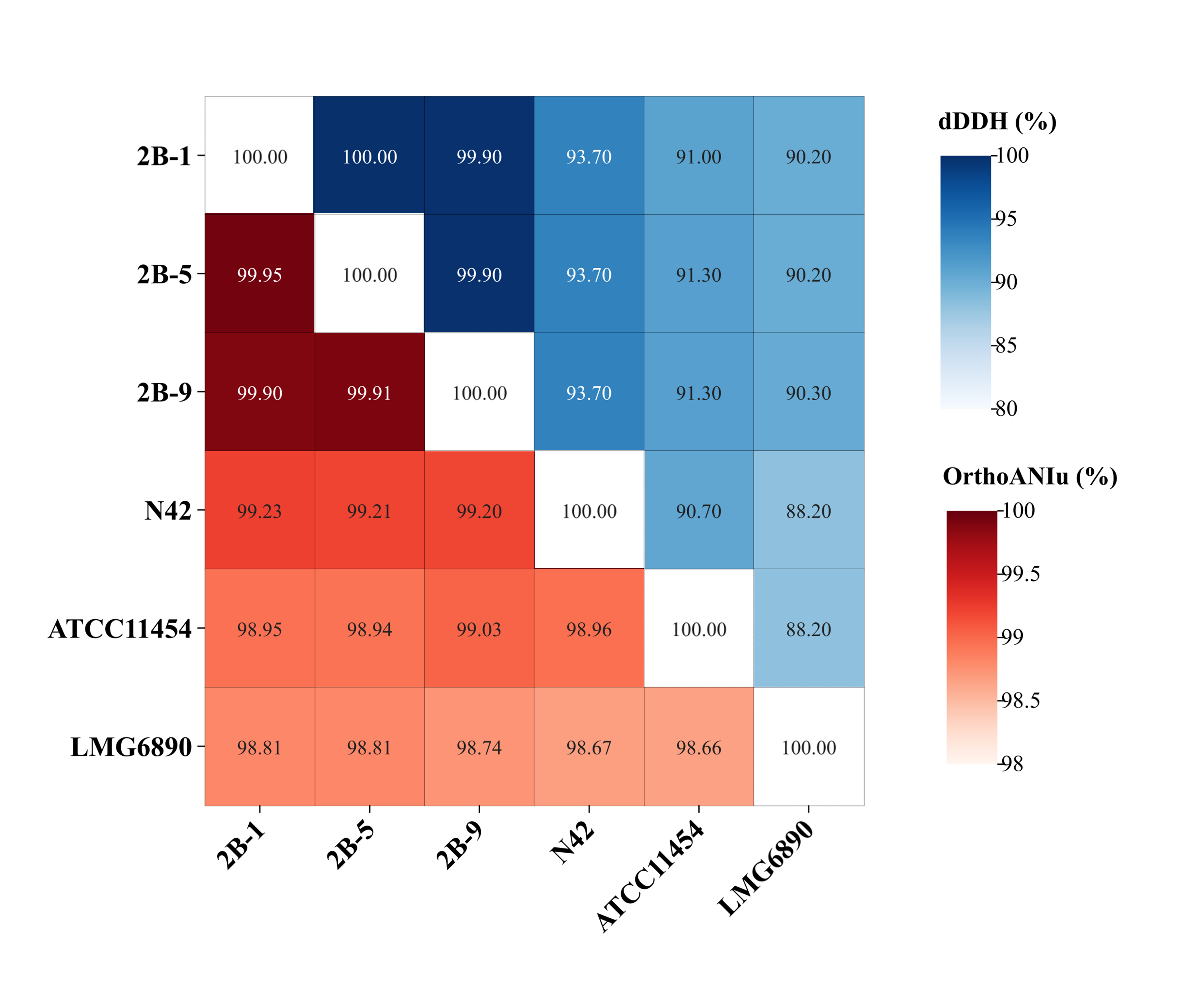


**Figure S1.** Heatmap displaying dDDH (upper right) and OrthoANIu (lower left) values between genome sequences of *L. lactis* subsp. *lactis* strains, including three freshwater isolates from this study and three strains retrieved from the Genbank (Table S2).


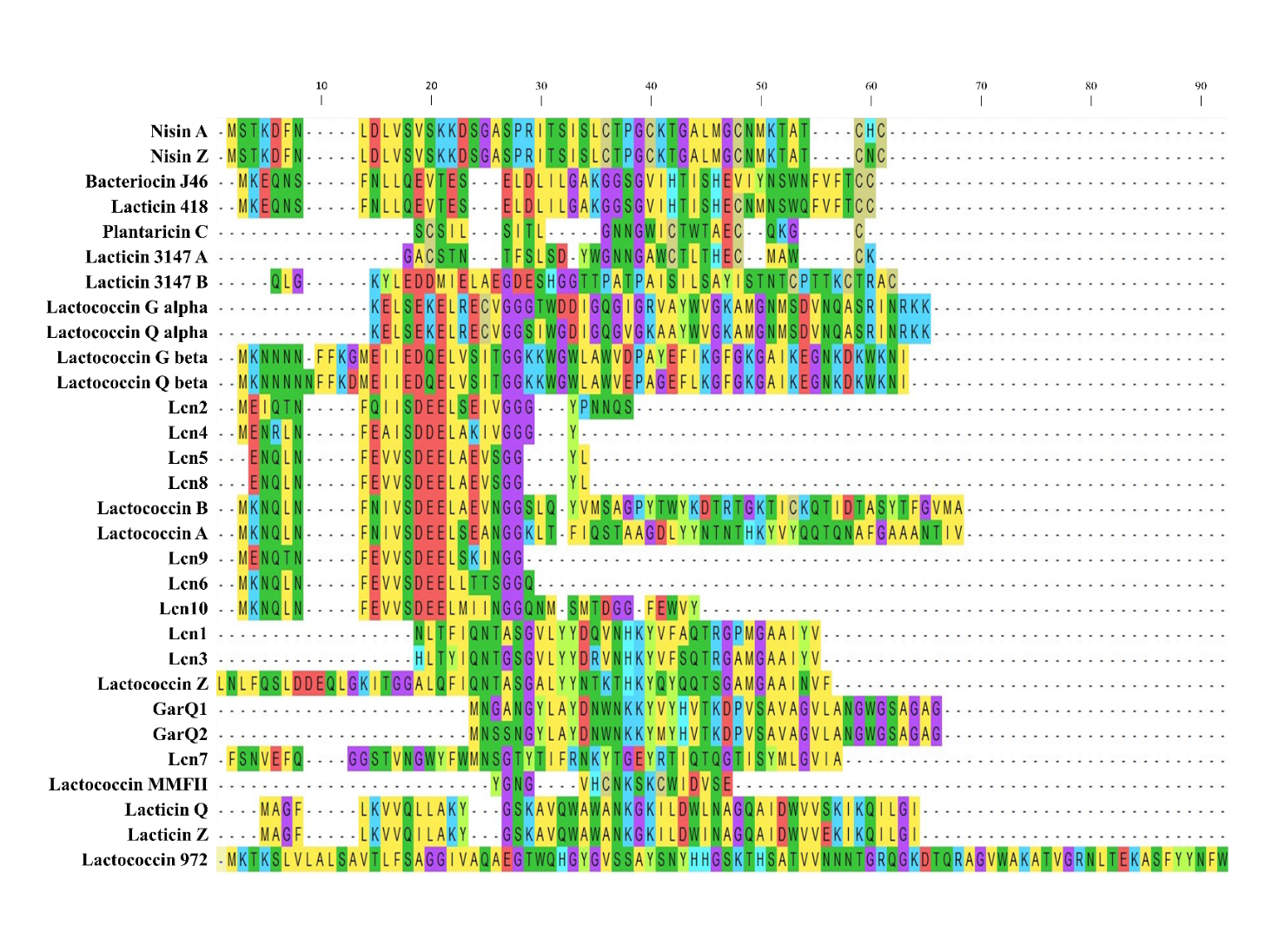


**Figure S2.** Amino acid sequence alignment of the detected bacteriocin domains from the analyzed genomes in this study with the domains of other known bacteriocins from GenBank (see Table S3 for detailed accession numbers).


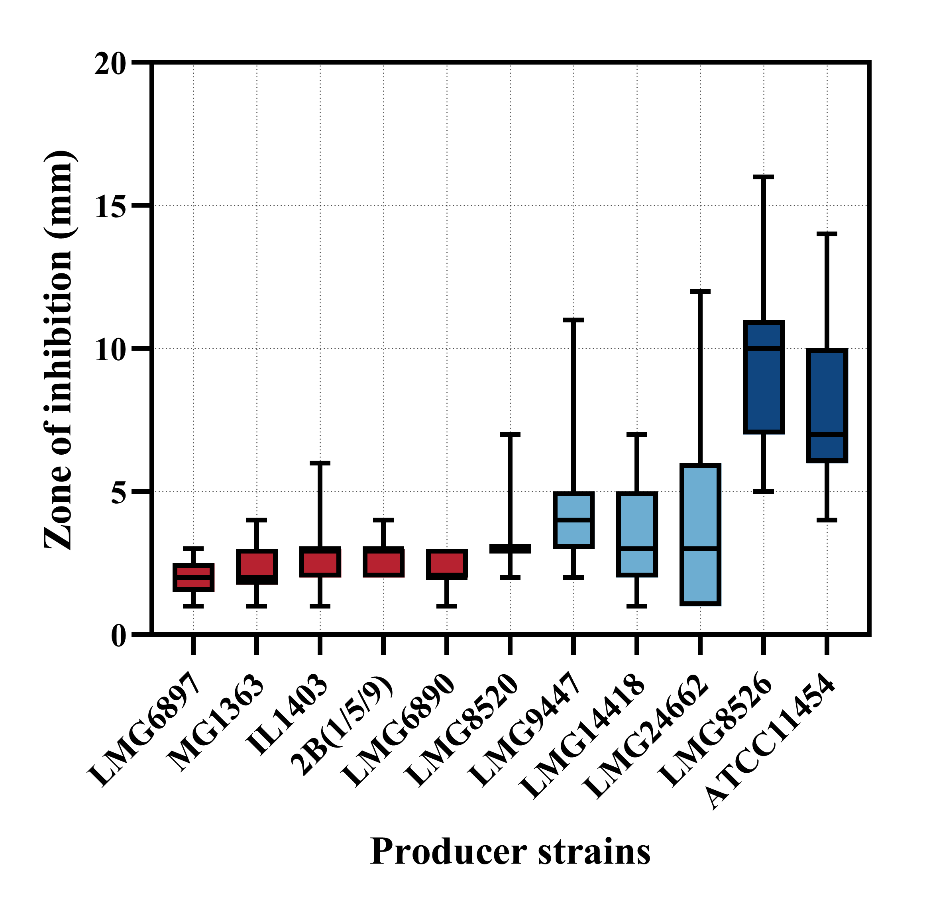


**Figure S3**. Antagonistic activity of the selected *L. lactis* subsp. *lactis* strains. The mean value and distribution of radius of growth inhibition zones of producer strains against various target strains were calculated and plotted. Different colors indicate different antagonism profiles.
